# Immunohistopathological Findings of Severe Cutaneous Adverse Drug Reactions

**DOI:** 10.1155/2017/6928363

**Published:** 2017-10-31

**Authors:** Mari Orime

**Affiliations:** Division of Dermatology, Niigata University Graduate School of Medical and Dental Sciences, 1-757 Asahimachi-dori, Chuo-ku, Niigata 951-8510, Japan

## Abstract

Diagnosis of severe cutaneous adverse drug reactions should involve immunohistopathological examination, which gives insight into the pathomechanisms of these disorders. The characteristic histological findings of erythema multiforme (EM), Stevens–Johnson syndrome (SJS), and toxic epidermal necrolysis (TEN) provide conclusive evidence demonstrating that SJS/TEN can be distinguished from EM. Established SJS/TEN shows full-thickness, extensive keratinocyte necrosis that develops into subepidermal bullae. Drug-induced hypersensitivity syndrome (DIHS) and exanthema in drug reaction with eosinophilia and systemic symptoms (DRESS) each display a variety of histopathological findings, which may partly correlate with the clinical manifestations. Although the histopathology of DRESS is nonspecific, the association of two or more of the four patterns—eczematous changes, interface dermatitis, acute generalized exanthematous pustulosis- (AGEP-) like patterns, and EM-like patterns—might appear in a single biopsy specimen, suggesting the diagnosis and severe cutaneous manifestations of DRESS. Cutaneous dendritic cells may be involved in the clinical course. AGEP typically shows spongiform superficial epidermal pustules accompanied with edema of the papillary dermis and abundant mixed perivascular infiltrates. Mutations in *IL36RN* may have a definite effect on pathological similarities between AGEP and generalized pustular psoriasis.

## 1. Introduction

Typical cutaneous adverse drug reactions (cADRs), such as maculopapular eruptions (MPEs), often show varying degrees of vacuolar interface dermatitis associated with nonspecific eosinophilic and/or neutrophilic infiltrates [1]. Nonetheless, the histopathologies of most of the severe cADRs are unique to each condition. The following reviews the immunohistopathological features of several severe cADRs.

## 2. Stevens–Johnson Syndrome (SJS)/Toxic Epidermal Necrolysis (TEN)

**Figure 1:**
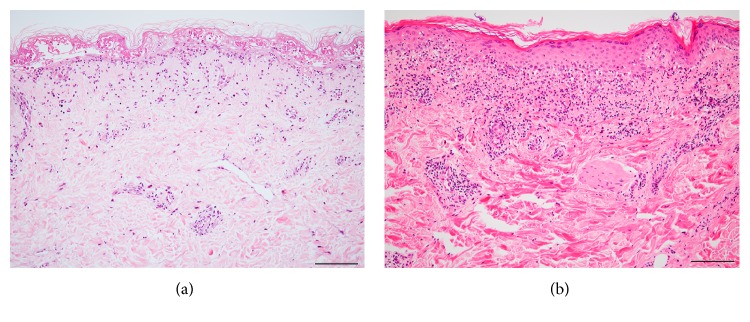
Hematoxylin-eosin (HE) sections of toxic epidermal necrolysis (TEN) (a) and erythema multiforme (EM) (b). (a) Subepidermal bullae under full-thickness epidermal necrosis. Note: the cell-poor dermal inflammation. (b) An interface reaction pattern with infiltrates of lymphocytes and scattered necrotic keratinocytes. Lymphocyte infiltrates are much denser in EM than in TEN. Bar = 100 *μ*m.

The general histological findings of SJS/TEN are subepidermal bullae with overlying confluent necrosis of the epidermis and a few perivascular lymphocytic infiltrates (Figure 1(a)) [2]. In the early stages of SJS/TEN, scattered necrotic keratinocytes appear in the lower layer of the epidermis, histologically resembling a feature of erythema multiforme (EM) major: necrotic keratinocytes spread around the epidermis with vacuolization at the epidermal-dermal junction (Figure 1(b)) [3, 4]. In established SJS/TEN, extensive full-thickness keratinocyte necrosis is seen, which results in the formation of subepidermal bullae. The epidermis exhibits major epidermal necrosis in SJS/TEN, whereas in EM major, the epidermis exhibits less necrosis, with changes appearing predominantly in the basal layer. The Japanese diagnostic criteria for SJS/TEN propose that at least ten necrotic keratinocytes be seen at a magnification of 200x. In the upper dermis, perivascular inflammatory infiltrates and exocytosis are minimal to absent. SJS/TEN tends to show less dermal inflammation than is seen in the pronounced dermal infiltration and extravasation of erythrocytes in EM major [5, 6]. By contrast, the degree of inflammation was shown in a study of 37 TEN patients to correlate with a worse prognosis, with the quantification of dermal mononuclear cell infiltration approximately as accurate as the TEN-specific severity-of-illness score (SCORTEN) in predicting patient outcome [7].

In SJS/TEN patients showing EM-like lesions, the initial diagnosis and prediction of disease activity can benefit from information gleaned from snap-frozen, immediately cryostat-sectioned hematoxylin and eosin-stained skin specimens [8].

Differential diagnoses other than EM major include staphylococcal scalded skin syndrome (SSSS), linear immunoglobulin A (IgA) bullous dermatosis, acute graft-versus-host disease (GVHD), and generalized bullous fixed drug eruption (GBFDE). SSSS displays only superficial, rather than full-thickness, epidermal necrosis, and the pathogenesis is staphylococcal exfoliative toxins that cleave a specific peptide bond on desmoglein 1 [9]. Linear IgA bullous dermatosis can be clinically similar to TEN, although the former shows no necrotic epidermis [10–12]. Complete epidermal necrosis may point to the need to distinguish severe acute GVHD from TEN. The most conspicuous epidermal change of acute GVHD is satellite cell necrosis comprising apoptotic keratinocytes adjacent to lymphocytes in the epidermis; however, when the epidermal necrosis is prominent, it can be hard to distinguish between the two diseases [13]. If the early exanthema of acute GVHD displays erythematous follicular papules showing folliculotropic infiltrates accompanied by basal vacuolization and satellite cell necrosis, the papules might help distinguish severe acute GVHD from TEN [14]. GBFDE also displays apoptotic keratinocytes throughout the epidermis, whereas infiltrating eosinophils and dermal melanophages are more frequently found in GBFDE than in SJS/TEN. Compared with SJS/TEN, the dermal CD4^+^ T cells, including Foxp3^+^ regulatory T cells, infiltrate to a greater extent in GBFDE. Additionally, both serum granulysin levels and the number of intraepidermal granulysin-expressing cells are much lower in GBFDE [15].

## 3. Drug-Induced Hypersensitivity Syndrome (DIHS)/Exanthema in Drug Reaction with Eosinophilia and Systemic Symptoms (DRESS)

Histopathological investigation is not critical for the diagnosis of DIHS according to diagnostic criteria established by a Japanese consensus group [16, 17], nor is it critical for the diagnosis of DRESS according to diagnostic criteria proposed by the European registry of severe cutaneous adverse reaction to drugs group (EuroSCAR/RegiSCAR) [16].

**Figure 2:**
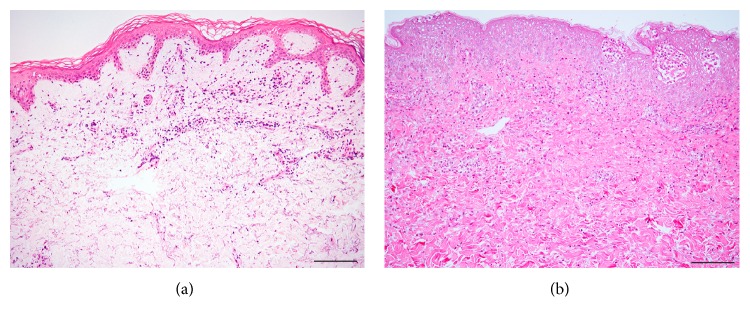
HE sections of drug-induced hypersensitivity syndrome/exanthema in drug reaction with eosinophilia and systemic syndrome. Two cases that are associated with liver function deficiency show different histopathologies: intermittent interface change, few necrotic keratinocytes, and slight spongiosis in (a); diffuse interface change, several necrotic keratinocytes, and considerable spongiosis with spongiotic bullae in (b). Bar = 100 *μ*m.

The heterogeneous histopathology of DRESS entails no specific diagnostic feature. Frequently reported findings include spongiosis, various degrees of basal vacuolization, necrotic keratinocytes, dense and diffuse dermal-epidermal infiltrates with lymphocytic exocytosis, dermal edema, and superficial perivascular infiltrates of mostly lymphocytes with or without eosinophils (Figures 2(a) and 2(b)) [18–20]. Clinicopathological investigations of DRESS have suggested that an association between two or more of four patterns—eczematous alterations, interface dermatitis, acute generalized exanthematous pustulosis- (AGEP-) like pattern, and EM-like pattern—in a single biopsy specimen may lead to the diagnosis and suggest the risk of severe cutaneous manifestations. These characteristics are remarkably more prominent in DRESS cases than in MPE cases [21]. Apoptotic keratinocytes have been shown to be more closely related to liver and/or renal complications [21–24]. Additionally, a recent study has demonstrated a close relationship between interface changes and cholestatic-type liver injury, which might imply an immunoallergic reaction in cholestatic-type liver injury in DRESS [25]. The intensity of the dermal lymphocytic infiltrates could correlate with DRESS severity [26]. Conversely, epidermal spongiosis correlates with the absence of renal complications and with nonsevere forms of DRESS [23]. Immunohistochemically, the number of plasmacytoid dendritic cells, a subset of leukocytes with the ability to produce interferon-*α* upon viral infection, increases in DIHS skin, and the number of these cells in the peripheral blood is diminished around the viral reactivation period [27]. Thymus and activation-regulated chemokine (TARC), a family of CC chemokines known to be vital for Th2-type immune response and to potentially reflect the activity of skin eruptions in DRESS, is expressed on CD11c^+^ dendritic cells in the dermis of the lesion site [28]. This indicates that such cells may be a major cause of TARC in DRESS [28].

The clinical features of SJS/TEN and AGEP may be similar to those of DRESS [29, 30]. However, the histopathology of DRESS differs substantially from that of TEN and AGEP; DRESS presents neither full-thickness necrosis nor sterile subcorneal pustules [31–33]. In our clinical experience, none of the following have been found to associate with DRESS severity: interface dermatitis, spongiosis, the degree of necrotic keratinocytes, and vascular damage (unpublished data). A recent publication showed that the coexistence of three patterns—eczematous, vascular, and interface dermatitis—was frequently observed in definite DRESS cases with high grades of cutaneous and hematological abnormalities [34]. The differences between our observations and those of this study might be due to our smaller sample. Differences in DRESS case definitions and the skin lesions' stages of evolution may account for the differences observed among diverse case reports and clinical studies [2]. The various clinical appearances, such as MPE-like and EM-like eruptions, might be responsible for the wide variety of histopathological findings observed in DRESS patients. In performing biopsies, it is recommended that the type of biopsy lesion—that is, macular or confluent erythema, purpura, papule, or pustule—be described in detail, for more than one area, and at several points in time. The relation between the onset of the skin eruption and the time of biopsy should be mentioned in terms of hours or days, instead of “early” or “late.”

The reactivation of several viruses, such as human herpesvirus- (HHV-) 6, HHV-7, cytomegalovirus (CMV), and Epstein-Barr virus, sometimes occurs over the prolonged clinical course [35]. Cutaneous lesions emerging as late systemic manifestations of CMV tend to be rare, presenting as ulcerated erythematous papules that histopathologically exhibit intranuclear inclusion [36]. Because cutaneous manifestations are associated with fatal gastrointestinal complications, early identification of CMV reactivation is crucial for effective management.

## 4. Acute Generalized Exanthematous Pustulosis (AGEP)

**Figure 3:**
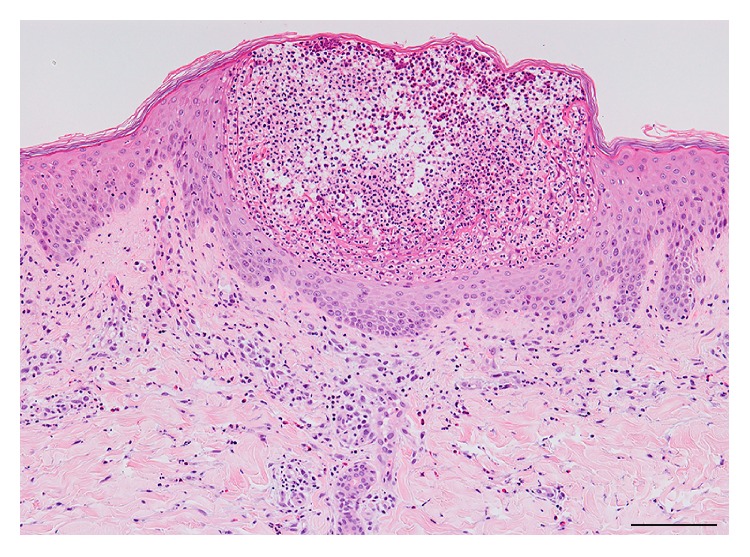
An HE section of acute generalized exanthematous pustulosis shows spongiform superficial intraepidermal pustules and polymorphous perivascular infiltrates containing mostly neutrophils. Bar = 100 *μ*m.

The histopathology of AGEP is typically spongiform subcorneal and/or superficial intraepidermal pustules accompanied with edematous papillary dermis and large amounts of perivascular infiltrates (Figure 3) [37, 38]. A large series of AGEP cases revealed several unique features: a higher prevalence of necrotic keratinocytes (67%), which was described as a major epidermal feature, and a conspicuously high prevalence of dermal infiltrates (93–100%) containing neutrophils (100%) as well as eosinophils (81%) [31]. The prevalence of leukocytoclastic vasculitis ranges from less than 1% to 20% of cases [39]. This difference might be attributed to misinterpreting erythrocyte extravasation as vasculitis [31].

AGEP and generalized pustular psoriasis (GPP) share common clinical manifestations: diffuse pustules over the entire body and systemic symptoms of high fever and neutrophil-predominant hyperleukocytosis [39]. Morphology of the spongiotic pustules is indistinguishable between that seen in AGEP or the acute phase of GPP. In one study of 43 cases of AGEP and 24 cases of GPP, AGEP was successfully differentiated from GPP by necrotic keratinocytes, mixed neutrophil-rich interstitial and middermal perivascular infiltrates, the presence of eosinophils in the pustules or dermis, and the absence of tortuous or dilated blood vessels. Furthermore, chronic GPP with pustules on prolonged existing lesions displays significant epidermal psoriasiform changes, such as hyperkeratosis and parakeratosis [32]. These pathological similarities between AGEP and GPP might stem from a mutually occurring mutation in *IL36RN* encoding the interleukin-36 receptor antagonist. Several cases of patients with AGEP with homozygous or heterozygous *IL36RN* mutations have been reported, particularly in patients presenting with intraoral involvement, which might underlie the defect in some forms of AGEP [40–42].

## 5. Conclusion

SJS/TEN might present particular histopathological findings if the condition is because of viral infection. Secondary cutaneous eruptions following immune checkpoint blockade therapy appear to show many histological findings distinct from those of classic cADRs [43].

Evaluating the histopathological features of these diseases, in combination with their severity, can lead to accurate diagnoses.
